# Aldehyde dehydrogenase 3B2 promotes the proliferation and invasion of cholangiocarcinoma by increasing Integrin Beta 1 expression

**DOI:** 10.1038/s41419-021-04451-8

**Published:** 2021-12-14

**Authors:** Yue Wang, Kangshuai Li, Wei Zhao, Zengli Liu, Jialiang Liu, Anda Shi, Tianli Chen, Wentao Mu, Yunfei Xu, Chang Pan, Zongli Zhang

**Affiliations:** 1 Department of General Surgery, Qilu Hospital, Cheeloo College of Medicine, Shandong University, 107 Wenhuaxi Road, 250012 Jinan, China; 2 Department of Emergency Medicine, Qilu Hospital, Cheeloo College of Medicine, Shandong University, 107 Wenhuaxi Road, 250012 Jinan, China; 3 Shandong Provincial Clinical Research Center for Emergency and Critical Care Medicine, Institute of Emergency and Critical Care Medicine of Shandong University, Chest Pain Center, Qilu Hospital, Cheeloo College of Medicine, Shandong University, 107 Wenhuaxi Road, 250012 Jinan, China; 4 Key Laboratory of Emergency and Critical Care Medicine of Shandong Province, Key Laboratory of Cardiopulmonary-Cerebral Resuscitation Research of Shandong Province, Qilu Hospital, Cheeloo College of Medicine, Shandong University, 107 Wenhuaxi Road, 250012 Jinan, China

**Keywords:** Prognostic markers, Bile duct cancer

## Abstract

Aldehyde dehydrogenases (ALDHs) play an essential role in regulating malignant tumor progression; however, their role in cholangiocarcinoma (CCA) has not been elucidated. We analyzed the expression of ALDHs in 8 paired tumor and peritumor perihilar cholangiocarcinoma (pCCA) tissues and found that ALDH3B1 and ALDH3B2 were upregulated in tumor tissues. Further survival analysis in intrahepatic cholangiocarcinoma (iCCA, *n* = 27), pCCA (*n* = 87) and distal cholangiocarcinoma (dCCA, n = 80) cohorts have revealed that ALDH3B2 was a prognostic factor of CCA and was an independent prognostic factor of iCCA and pCCA. ALDH3B2 expression was associated with serum CEA in iCCA and dCCA, associated with tumor T stage, M stage, neural invasion and serum CA19-9 in pCCA. In two cholangiocarcinoma cell lines, overexpression of ALDH3B2 promoted cell proliferation and clone formation by promoting the G1/S phase transition. Knockdown of ALDH3B2 inhibited cell migration, invasion, and EMT in vitro, and restrained tumor metastasis in vivo. Patients with high expression of ALDH3B2 also have high expression of ITGB1 in iCCA, pCCA, and dCCA at both mRNA and protein levels. Knockdown of ALDH3B2 downregulated the expression of ITGB1 and inhibited the phosphorylation level of c-Jun, p38, and ERK. Meanwhile, knockdown of ITGB1 inhibited the promoting effect of ALDH3B2 overexpression on cell proliferation, migration, and invasion. ITGB1 is also a prognostic factor of iCCA, pCCA, and dCCA and double-positive expression of ITGB1 and ALDH3B2 exhibits better performance in predicting patient prognosis. In conclusion, ALDH3B2 promotes tumor proliferation and metastasis in CCA by regulating the expression of ITGB1 and upregulating its downstream signaling pathway. The double-positive expression of ITGB1 and ALDH3B2 serves as a better prognostic biomarker of CCA.

## Introduction

Cholangiocarcinoma (CCA) is a cancer type arising from the biliary duct which is associated with poor prognosis [1]. CCA accounts for about 3% of gastrointestinal cancers; however, the morbidity and mortality of cholangiocarcinoma, especially iCCA, are increasing worldwide [2–4]. According to the location, CCA is divided into intrahepatic cholangiocarcinoma (iCCA), perihilar cholangiocarcinoma (pCCA), and distant cholangiocarcinoma (dCCA), which also have different clinical features, treatment strategy, prognosis, and oncologic characters [5–7]. Currently, radical resection of the tumor is the only curative treatment for CCA. However, most patients are diagnosed with an advanced stage and lose the surgical opportunity, which makes the resectable rate of CCA relatively low [8–12]. The studies of biomarkers and drug targets of CCA are very rare, resulting in the slow processes of CCA targeted therapy. Hence, both effective pre-and post-operational biomarkers are urgently needed for early diagnosis and individualized treatment of CCA [13–15].

Aldehyde dehydrogenase *3B2(ALDH3B2)*, also known as *ALDH8*, belongs to the aldehyde dehydrogenase (*ALDH*) gene superfamily. [16] This superfamily consists of 19 putatively functional genes that encode enzymes catalyzing the oxidation of aldehydes to the corresponding carboxylic acids [17]. Mutations and polymorphism of ALDH genes, resulting in a decreased or lost enzymatic activity, could lead to severe diseases such as Sjögren-Larsson syndrome, cancer, and Alzheimer’s disease [18, 19]. The majority of ALDH proteins have been well characterized. However, little is known about the function of the *ALDH3B2* isozyme. One of the ALDH family members, ALDH1, was identified to be an important cancer stem cell marker in a series of malignant tumors [20–24]. Although ALDHs are found to play an important role in the progression of several malignant tumors and cardiovascular diseases [25–31], the expression patterns and differences in expression between tumor and peritumor tissues of cholangiocarcinoma have not been evaluated yet. Moreover, the expression and function of ALDH in CCA have never been studied.

In this study, the expression pattern of 19 ALDH family members in 8 paired pCCA tumor and peritumor tissues were first analyzed with mRNA sequencing and ALDH3B1 and ALDH3B2 was found upregulated in pCCA tissues. The result was further verified by qPCR. The expression of ALDH3B1 and ALDH3B2 in 27 iCCAs, 87 pCCAs, and 80 dCCAs was detected with immunohistochemistry (IHC), and the prognostic role of ALDH3B1 and ALDH3B2 were then analyzed with univariate and multivariate analyses. ALDH3B2 was overexpressed or knocked down in two CCA cell lines, and the in vitro and in vivo assays of ALDH3B2 on cell migration, invasion, and proliferation were also evaluated after regulating ALDH3B2 expression. Finally, the potential downstream signaling pathway of ALDH3B2 was analyzed and ITGB1 was identified as a target effector of ALDH3B2-induced CCA progression. Double-positive expression of ITGB1 and ALDH3B2 was identified as a better prognostic biomarker of CCA.

## Materials and methods

### Cell culture and transfection

The human normal bile duct cell line HIBEpiC and cholangiocarcinoma cell lines, QBC-939, RBE, and HCCC9810, were used in this study. The cell lines were all purchased from the Cell Bank of the Chinese Academy of Sciences (Shanghai, China), authenticated by STR analysis, and tested for mycoplasma contamination. Cells were cultured in DMEM culture medium supplemented with 10% fetal bovine serum (FBS)and 1% penicillin/streptomycin under 5% CO_2_ at 37 °C. ALDH3B2 knockdown was obtained by lentivirus transfection. The ALDH3B2 lentivirus and vector control were constructed by Shanghai Genechem Co., LTD. (Shanghai, China). The sequence of ALDH3B2-specific shRNA (CAGTACCTGGACCAGAGCTGCTTTGCCGT) was obtained from previous reports [32] and inserted into the GV493 vector. The ALDH3B2 overexpression plasmid was constructed by inserting the full length of human ALDH3B2 (gene ID: NM_001031615.3) into the TK-PCDH-copGFP-T2A-Puro vector. Cells were seeded into each well of a six-well plate at a density of 3 × 10^5^. The lentiviruses were diluted in 2 ml of DMEM supplementing with no FBS and 5 μg/ml polybrene (Sigma, USA) and added to the well. 24 h later, the medium containing the virus was replaced with a fresh medium containing 10% FBS. The knockdown clones were generated using pooled clone. ALDH3B2 expression was validated via qPCR and/or western blot.

### Patient and ethical approval

The patient cohort was previously reported [10, 33] and comprised of patients who underwent surgery for iCCA (27 cases), pCCA (87 cases), and dCCA (80 cases) at Qilu Hospital of Shandong University from 2012 to 2018. The patient cohorts were selected according to the following inclusion criteria: (i) patients who underwent radical resection with a clear surgical margin; (ii) patients with available formalin-fixed tumor tissues, follow-up information, and complete medical records; (iii) patients with no history of other malignancies. Fresh tumor tissues and tumor-adjacent normal tissues from 6 pairs of iCCA patients, 6 pairs of pCCA patients, and 6 pairs of dCCA patients were also collected continuously. The 8th AJCC/UICC TNM classification system was applied to classify and stage the tumors. Informed consent was obtained from all patients. All experiments involving human tissues and animals were approved by the Ethics Committee of Qilu Hospital of Shandong University.

### RNA interference

For knockdown of ITGB1, the cholangiocarcinoma cells were transfected using Opti-MEM medium and Lipofectamine (Invitrogen) according to the manufacturer’s instructions. Besides, the siRNAs were purchased from Genepharma. The siRNA sequence of ITGB1 is as follows: 5′-GCUCAGUCUUACUAAUAAATT-3′.

### RNA isolation and real-time qPCR

RNA isolation and real-time qPCR were performed as previously described [7, 34]. Real-time qPCR was performed on RNA extracted from the cholangiocarcinoma cell lines or tissues. Total RNA was isolated according to the manufacturer’s manual. The concentration and purity of the RNA samples were determined using the A260-A280 nm ratio. The following primers were used: ALDH3B2, 5′-TACTTCAATGCCGGCCAGAC-3′ (forward) and 5′-GATGATGTGGCCCAGGTTTG-3′ (reverse); GAPDH, 5′-GCACCGTCAAGGCTGAGAAC-3′ (forward) and 5′-TGGTGAAGACGCCAGTGGA-3′ (reverse); and ITGB1, 5′-AAAAGAAGGGTTGCCCTCCAG-3′ (forward) and 5′-GCTCCCCTGATCTTAATCGCA-3′ (reverse).

### Immunohistochemistry analysis

Immunohistochemistry (IHC)analysis was performed as previously described [5, 6]. Tissue microarray (TMA) was constructed using paraffin-embedded sections of tumors after hematoxylin and eosin staining to confirm the representative tumor area. Core tissues with a 1.5-mm diameter were used for TMA construction. IHC was performed on the TMA slides for the detection of ALDH3B1, ALDH3B2, and ITGB1. In brief, the slides were submerged in EDTA (pH = 8.0) buffer for optimal antigen retrieval. The tissue microarray slide was incubated with rabbit antibody anti-ALDH3B1 (1:50; proteintech, USA), anti-ALDH3B2 (1:50; proteintech, USA), and ITGB1 (1:200; Cell Signaling Technology, USA) at 4 °C overnight. A biotin-labeled goat anti-rabbit antibody (Zsbio, Beijing, China) was applied for 30 min at room temperature. Subsequently, the slides were incubated with conjugated horseradish peroxidase streptavidin. The peroxidase reaction was developed using a 3,3-diaminobenzidine (DAB) solution (Zsbio).

The IHC results were evaluated independently by two senior pathologists who were unaware of the clinical information. The IHC results were semi-quantitatively scored in a conventional way based on the staining intensity (0, negative; 1, weak; 2, moderate; 3, strong; refer to Supplemental Fig. 1 for the typical images of each staining intensity level) and the percentage of positively stained cells (0, 0%; 1, 1–25%; 2, 25–50%; 3, 50–75%; 4, 75–100%). The final score was the product of the two scores multiplied together.

### Western blot assays

Western blot assays were performed as previously described [11, 35]. Total protein was obtained using RIPA lysis buffer supplemented with protease inhibitors and phosphatase inhibitors from normal bile duct cell line or cholangiocarcinoma cell lines. A total of 30 μg protein was subjected to western blot and then separated using 10% SDS-PAGE. The SDS-PAGE was then electrotransferred onto the polyvinylidene difluoride membranes. Membranes were blocked using 5% BSA and then subjected to incubation with the primary antibody. The mouse antibody for GAPDH was purchased from Santa Cruz. The rabbit antibodies for ALDH3B2 were purchased from proteintech. The rabbit antibodies for E-cadhesion N-cadhesion, snail, ITGB1, p-c-Jun, c-Jun, p-p38, p38, p-ERK, and total ERK were purchased from CST. Information of the antibodies applied in this study has been summarized in Supplemental Table 1.

### Wound healing assay

For the wound healing assay, control cells, ALDH3B2 knockdown cells, scramble cells, ALDH3B2-overexpressing cells, and vector cells were plated in 6-well plates separately. After complete attachment, a sterile tip was used to draw straight lines on the bottom of the plate. The plates were then washed with PBS for 3 times. The initial wound size was also recorded with a microscope afterward. After incubation at 37 °C for 24 h, the wound size was re-recorded. The wound closure was calculated according to the following formula: (1-[current wound size/initial wound size]) × 100.

### Cell migration and invasion assay

Cell migration or invasion was evaluated using transwell chambers coated without or with matrigel. In brief, a total of 1 × 10^5^ cells were seeded into the upper chamber of the 8.0-μm pore polycarbonate membrane for adhesion without or with a monolayer of 5% Matrigel. The chambers were then put in one pore of the 24-well plates containing medium with 10% FBS at the bottom. After incubation at 37 °C for 24 h (RBE cells) or 48 h (QBC939 cells), the cells migrating to the lower surface of the chamber were fixed with methanol and then stained with 0.1% crystal violet solution (Sigma-Aldrich). Three visual fields were selected and the number of cells in the membrane was counted.

### CCK8 assay

CCK8 assays were performed as previously described [35]. 200 μl of medium containing three thousand cells were seeded into each pore of a 96-well plate, and cell proliferation was measured 6, 24, 48, and 72 h after seeding according to the manufacturer’s protocol. In brief, cells were incubated with 100 μl of reaction mixture containing 10 μl CCK-8 and 90 μl DMEM for 2 h and then measured at a wavelength of 450 nm.

### Clone formation assay

Clone formation assays were performed as previously described [35]. One thousand cells were seeded into a six-well plate and then cultured under 5% CO_2_ at 37 °C for 2 weeks (QBC939 cells) or 4 weeks (RBE cells). The colonies were fixed with formalin for 30 min and then stained with 0.1% crystal violet for 15 min.

### Flow cytometry

Flow cytometry analysis was performed as previously reported [33, 35]. For cell cycle analysis, the ALDH3B2 knockdown cells, ALDH3B2-overexpressing cells, control cells, vector cells, and the scramble cells were harvested and fixed with cold 75% ethyl alcohol overnight at −20 °C. The cells were then washed with PBS and gently resuspended in 500 μl of propidium iodide (PI)/RNase staining solution (BD Biosciences, Franklin Lakes, NJ, USA) and incubated at 4 °C for 30 min in the dark. The samples were finally analyzed using Modifit software (Verity Software House, USA). For apoptosis analysis, cells were washed with phosphate-buffered saline (PBS) and stained with annexin V and 7-AAD (BD Pharmingen, San Diego, CA, USA). Fluorescence was measured using a FACSCalibur (BD Biosciences, San Jose, CA, USA) and analyzed using FlowJo (Tree Star, Ashland, OR, USA).

### In vivo live imaging assay

All animal experiments were approved by the Medical Ethics Committee of Shandong University. For the in vivo metastasis assay, 5 × 10^5^ RBE cells with ALDH3B2 knockdown were injected into the caudal vein of each nude mouse. The nude mouse was separated into two groups randomly and each group contain 6 mice. The tumor metastases were monitored by a live imaging system (IVIS Spectrum). The mice weights were measured every week, and the weights of livers were measured to assess the actual tumor burden. The number of nodules on the livers and lungs was confirmed by HE staining and counted.

### Statistical analysis

Statistical analysis was performed using SPSS 25.0 for Windows (SPSS Inc., Chicago, IL, USA). The expression of continuous variables is in the form of mean ± SD or median (interquartile range). Categorical variables were analyzed using the χ^2^ test or Fisher’s exact test, and continuous variables were analyzed using Student’s t-test. Univariate survival analysis was finished with the Kaplan–Meier (K–M) method. The relative prognostic significance of the variables for overall survival (OS) was evaluated using Cox proportional hazards regression models. All statistical tests were two-tailed, and *p* < 0.05 was considered significant. GraphPad Prism 8 (GraphPad Software, San Diego, CA, USA) was also used for statistical analysis.

## Results

### Identification of ALDH3B2 as a prognostic factor of cholangiocarcinoma

**Fig. 1:**
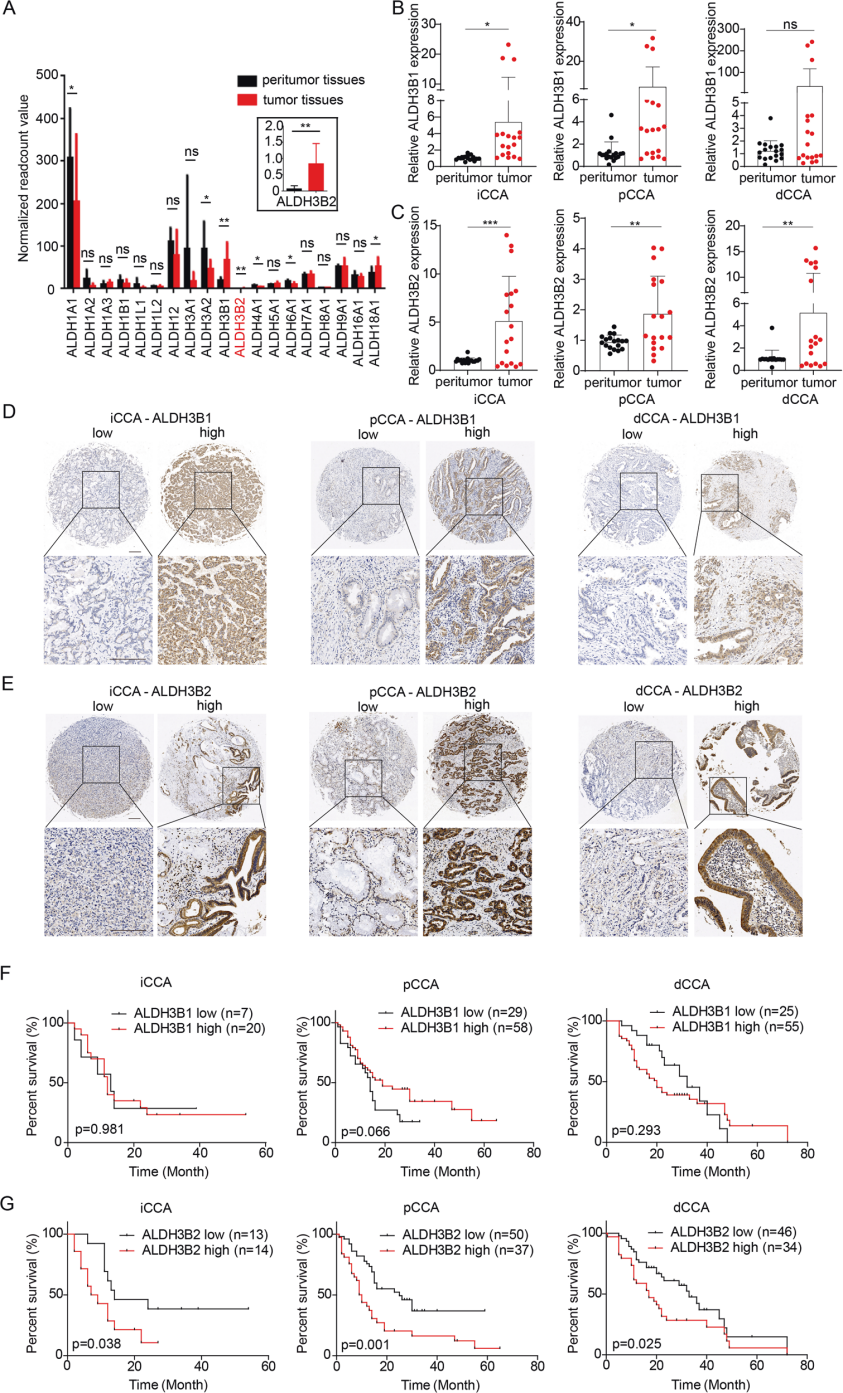
Expression and prognostic role of ALDH3B1 and ALDH3B2 in CCA. **A** Normalized read count value was employed to indicate the expression of 19 ALDH family members in 8 paired peritumor and pCCA tumor tissues (GSE139048 and GSE132279). ALDH3B1 and ALDH3B2 were upregulated in tumor tissues compared to peritumor tissues. Statistical significance between groups was assessed using paired t-test. ns represents not significant, and * represents *p* < 0.05, ** represents *p* < 0.01. **B** Relative mRNA expression of ALDH3B1 was quantified by qRT-PCR for 6 cases of iCCA (left), pCCA (middle), and dCCA (right) and their patient-paired normal tissues. The results were analyzed using the 2^−ΔΔCT^ method with GAPDH as a reference gene. Statistical significance between groups was assessed using the Student t-test. ns represents not significant, and * represents *p* < 0.05, ** represents *p* < 0.01, *** represents *p* < 0.001. **C** Relative mRNA expression of ALDH3B2 was quantified by qRT-PCR for 6 cases of iCCA (left), pCCA (middle), and dCCA (right) and their patient-paired normal tissues. The results were analyzed using the 2^−ΔΔCT^ method with GAPDH as a reference gene. Statistical significance between groups was assessed using the Student t-test. ns represents not significant, and * represents *p* < 0.05, ** represents *p* < 0.01, *** represents *p* < 0.001. **D**, **E** Representative images of immunohistochemical staining for ALDH3B1 (**D**) and ALDH3B2 (**E**) in iCCA (left), pCCA (middle), and dCCA (right) in the tissue microarray. **F**, **G** The overall survival curves of patients with iCCA (left), pCCA (middle), and dCCA (right) were stratified by ALDH3B1 (**F**) or ALDH3B2 (**G**) expression. High ALDH3B2 expression indicated poor patient overall survival.

We first analyzed the expression differences of 19 ALDH family members by mRNA sequencing between the tumor and paired tumor-adjacent tissues in 8 pCCA patients (GSE139048 and GSE132279). Most ALDHs showed no difference between tumor and peritumor tissues. And only *ALDH1A1, ALDH3A2, ALDH3B1, ALDH3B2, ALDH4A1, ALDH6A1*, and *ALDH18A1* were differentially expressed. Among these differentially expressed ALDHs, ALDH3B1 and ALDH3B2 were highly expressed in tumor tissues compared with the peritumor tissues with the largest fold of changes (Fig. 1A). Therefore, we selected ALDH3B1 and ALDH3B2 for validation.

The expression of *ALDH3B1* and *ALDH3B2* was then verified with qPCR in paired tumor and peritumor iCCA, pCCA, and dCCA tissues (*n* = 6, Fig. 1B, C). In consistent with the mRNA sequencing results, both *ALDH3B1* and *ALDH3B2* mRNAs were elevated in iCCA, pCCA, and dCCA compared with their corresponding adjacent tissues. In addition, the expression and intracellular localization of *ALDH3B1* and *ALDH3B2* were detected with IHC in 194 CCAs including 27 iCCAs, 87 pCCAs, and 80 dCCAs. The patients were divided into different subsets with low or high expression of ALDH3B1/ALDH3B2(Fig. 1D, E).

The correlations between ALDH3B1, ALDH3B2 and the overall survival rates were plotted by the Kaplan-Meier method. Interestingly, ALDH3B1 had no significant associations with OS rate (Fig. 1F), while ALDH3B2 was negatively correlated with prognosis in iCCA (*p* = 0.038), pCCA (*p* = 0.001) and dCCA (*p* = 0.025) (Fig. 1G). The 3-year OS of patients with low and high ALDH3B2 in iCCA, pCCA and dCCA were 38.5% vs. 10.7%, 36.9% vs. 16.3% and 42.3% vs. 28.5%, respectively. Associations between ALDH3B2 expression and clinicopathologic characteristics of CCA were further evaluated with the chi-square test. Patients with high ALDH3B2 expression were more likely to have advanced T stage and M stage, positive neural invasion and high serum CA19-9 in pCCA, while high ALDH3B2 expression was associated with high serum CEA in iCCA or dCCA (Table 1).

**Table 1:** Correlations between ALDH3B2 expression and clinicopathological characteristics in CCA.

Characteristics	Category	iCCA		*p*	pCCA		*p*	dCCA		*p*
		ALDH3B2 low (*n* = 13)	ALDH3B2 high (*n* = 14)		ALDH3B2 low (*n* = 50)	ALDH3B2 high (*n* = 37)		ALDH3B2 low (*n* = 46)	ALDH3B2 high (*n* = 34)	
Age (years)	<60	10	6	0.120	14	12	0.655	17	7	0.114
	≥60	3	8		36	25		29	27	
Gender	male	7	9	0.704	36	27	0.920	29	23	0.670
	female	6	5		14	10		17	11	
Tumor size (cm)	<3	3	4	1.000	32	16	0.054	36	26	0.850
	≥3	10	10		18	21		10	8	
Differentiation	Well/Moderate	6	10	0.252	34	20	0.185	22	21	0.216
	Poor	7	4		16	17		24	13	
T stage	T1/T2	10	10	1.000	50	33	0.030*	25	14	0.244
	T3/T4	3	4		0	4		21	20	
N stage	N0	11	12	1.000	35	23	0.443	31	24	0.760
	N1/N2	2	2		15	14		15	10	
M stage	M0	12	11	0.596	47	29	0.048*	45	32	0.388
	M1	1	3		3	8		1	2	
TNM stage	I-II	10	10	1.000	36	24	0.477	42	31	0.984
	III-IV	3	4		14	13		4	3	
Neural invasion	Negative	10	12	0.648	27	10	0.012*	22	12	0.262
	Positive	3	2		23	27		24	22	
CEA	<10	12	7	0.033*	44	33	1.000	46	29	0.012*
(ng/ml)	≥10	1	7		6	4		0	5	
CA19-9	<100	6	5	0.704	28	9	0.003*	25	14	0.244
(U/ml)	≥100	7	9		22	28		21	20	

*ALDH3B2* aldehyde dehydrogenase 3B2, *CCA* cholangiocarcinoma.

Univariate and multivariate analysis was then performed to identify the independent prognostic factors of CCA (Table 2). ALDH3B2 was confirmed as the independent prognostic biomarker of iCCA(*p* = 0.047) and pCCA(*p* = 0.042) indicating the unfavorable prognosis. In dCCA, ALDH3B2 tended to predict a poor prognosis of dCCA with an insignificant statistical significance (*p* = 0.096).

**Table 2:** The prognostic significance of ALDH3B2 and clinicopathological characteristics in CCA.

Characteristics	iCCA				pCCA				dCCA			
	Univariate analysis		Multivariate analysis		Univariate analysis		Multivariate analysis		Univariate analysis		Multivariate analysis	
	3-year OS	*p*^a^	HR	*p*^b^	3-year OS	*p*^a^	HR	*p*^b^	3-year OS	*p*^a^	HR	*p*^b^
*Age (years)*												
<60	36.5	0.132			41.9	0.029	1		45.4	0.127		
≥60	9.1				21.6		2.355	0.009	32.6			
*Gender*												
male	31.3	0.794			32.6	0.358			30.7	0.364		
female	18.2				20.5				47.2			
*Tumor size*												
<3 cm	14.3	0.246			33.8	0.157			37.9	0.131		
≥3 cm	28.6				21.0				29.6			
*Differentiation*												
Well/moderate	23.4	0.710			32.3	0.051	1		41.0	0.554		
Poor	27.3				21.9		1.874	0.036	29.3			
*T stage*												
T1/T2	34.3	0.032	1		29.7	0.014	1		47.8	0.009	1	
T3/T4	0		2.708	0.041	0		3.822	0.022	26.6		1.648	0.084
*N stage*												
N0	29.3	0.083	1		40.4	0.003	1		39.3	0.032	1	
N1/N2	0		0.890	0.885	5.5		1.381	0.334	30.9		1.418	0.256
*M stage*												
M0	29.8	0.252			33.7	<0.001	1		38.0	<0.001	1	
M1	0				0		2.788	0.008	0		3.588	0.048
*TNM stage*												
I/II	34.3	0.032			37.5	0.004			36.4	0.204		
III/IV	0				7.1				42.1			
*Neural invasion*												
Negative	21.8	0.348			42.1	0.221			48	0.006	1	
Positive	40.0				19.3				28.3		1.994	0.021
*CEA (ng/ml)*												
<10	35.5	0.006	1		30.4	0.022	1		38.2	0.070	1	
≥10	0		2.003	0.214	0		2.630	0.027	0		1.817	0.292
*CA19-9 (U/mL)*												
<100	43.6	0.027	1		36.1	0.020	1		47.7	0.108		
≥100	12.5		2.619	0.054	22.6		1.182	0.586	25.8			
*ALDH3B2*												
Low	38.5	0.038	1		36.9	0.001	1		42.3	0.025	1	
High	10.7		2.542	**0.047**	16.3		1.905	**0.042**	28.5		1.590	0.096

*ALDH3B2* aldehyde dehydrogenase 3B2, *CCA* cholangiocarcinoma, *OS* overall survival, *HR* hazard ratio.

^a^ Calculated by log-rank test.

^b^ Calculated by Cox-regression Hazard model.

### ALDH3B2 promotes cell proliferation by promoting the transition of the cell cycle from G0/G1 phase to S phase of CCA cells

**Fig. 2:**
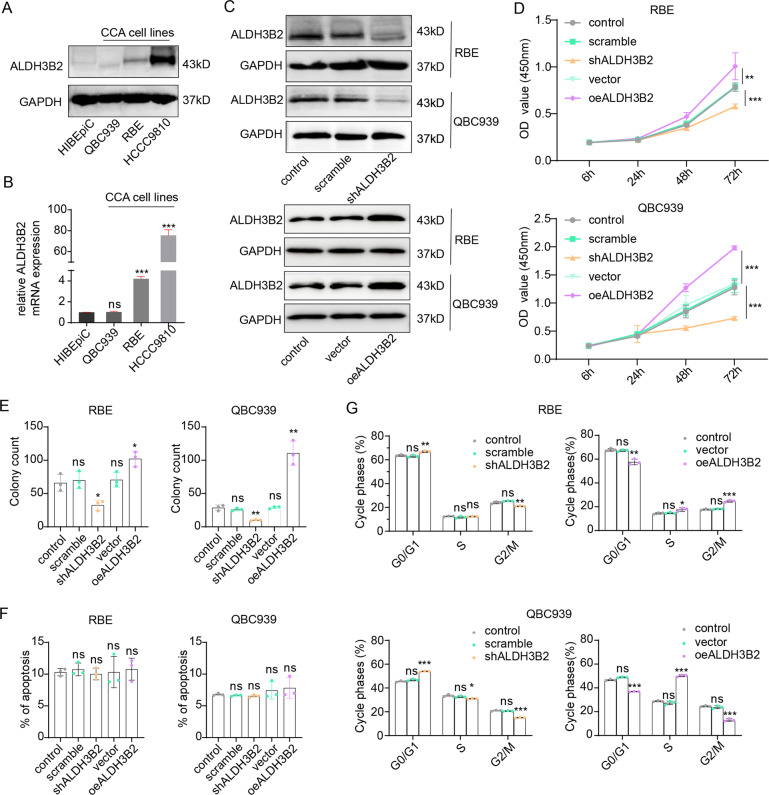
ALDH3B2 promotes the cell proliferation ability of CCA by promoting the G1/S phase transition in vitro. **A**, **B** Expression levels of ALDH3B2 in the human bile duct cell line HIBEpiC and CCA cell lines QBC939, RBE, and HCCC9810 were detected by western blot (**A**) and qRT-PCR (**B**). ALDH3B2 expression was upregulated in RBE and HCCC9810 CCA cell lines. **C** Western blot analysis of the knockdown and overexpression efficiency of ALDH3B2 in RBE and QBC939 cells. ALDH3B2 was successfully knocked down (upper) or overexpressed (lower) in these two cell lines. **D** CCK-8 assays were performed to identify the alteration in cell proliferation ability after ALDH3B2 knockdown or overexpression in RBE (upper) and QBC939 (lower) cells. **E** Clone formation of cells with ALDH3B2 knockdown or overexpression compared with their vector controls in RBE (left) and QBC939 cells (right). Knockdown of ALDH3B2 inhibited cell clone formation, while overexpression of ALDH3B2 promoted cell clone formation in vitro. **F** Flow cytometry was performed for detecting apoptotic rates in ALDH3B2-overexpressing and knockdown RBE (left) and QBC939 cells (right) cells. ALDH3B2 did not affect the apoptotic rates in these two cell lines. ns: no significance. **G** The effects of ALDH3B2 on the cell cycle were detected with flow cytometry after ALDH3B2 knockdown (left) or overexpression (right) in RBE (upper) and QBC939 cells (lower). Knockdown of ALDH3B2 inhibited the transition of the cell cycle from G1 to S phase, while overexpression of ALDH3B2 promoted the transition from G1 to S phase in vitro. ns: no significance; *p < 0.05; **p < 0.01; ***p < 0.001.

The intracellular function of ALDH3B2 was then investigated in CCA cell lines. Expression of ALDH3B2 in normal bile duct cell line HIBEpiC and cholangiocarcinoma cell lines including QBC-939, RBE and HCCC9810 were examined by Western Blot (Fig. 2A) and qPCR (Fig. 2B). ALDH3B2 was substantially upregulated in RBE and HCCC9810CCA cell lines than normal cell line (Fig. 2A, B). Stable RBE and QBC939 CCA cells with ALDH3B2 overexpression or knockdown were then established and validated via Western Blot (Fig. 2C).

CCK8 assays were performed to evaluate the effect of ALDH3B2 overexpression or knockdown on the cell proliferation ability of CCA (Fig. 2D). Downregulation of ALDH3B2 attenuated the proliferation ability of both RBE and QBC939 cell lines and ALDH3B2 overexpression extensively promoted the CCA proliferation. With clone formation assay, we showed that the clone formation ability of the ALDH3B2-silencing RBE and QBC939 cells were significantly weakened, while ALDH3B2 overexpression significantly promoted the colony formation ability of CCA cells (Fig. 2E, Supplemental Fig. 2A).

The effect of ALDH3B2 on the apoptosis rate of CCA cells was evaluated by flow cytometry. Knockdown or overexpression of ALDH3B2 did not alter the apoptosis rate of both RBE and QBC939 cell lines (Fig. 2F, Supplemental Fig. 2B). The effect of ALDH3B2 on the cell cycle was further analyzed via flow cytometry. Knockdown of ALDH3B2 increased the G1 phase ratio and decreased the S phase ratio both in the RBE and QBC939 cell lines, and ALDH3B2 overexpression had the opposite effects (Fig. 2G, Supplemental Fig. 2C). Taken in all, these results indicate that ALDH3B2 could promote CCA cell proliferation by inducing the transition of the cell cycle from G0/G1 phase to the S phase.

### ALDH3B2 promotes cell migration and invasion ability of CCA and induces epithelial-mesenchymal transition (EMT) in vitro

**Fig. 3:**
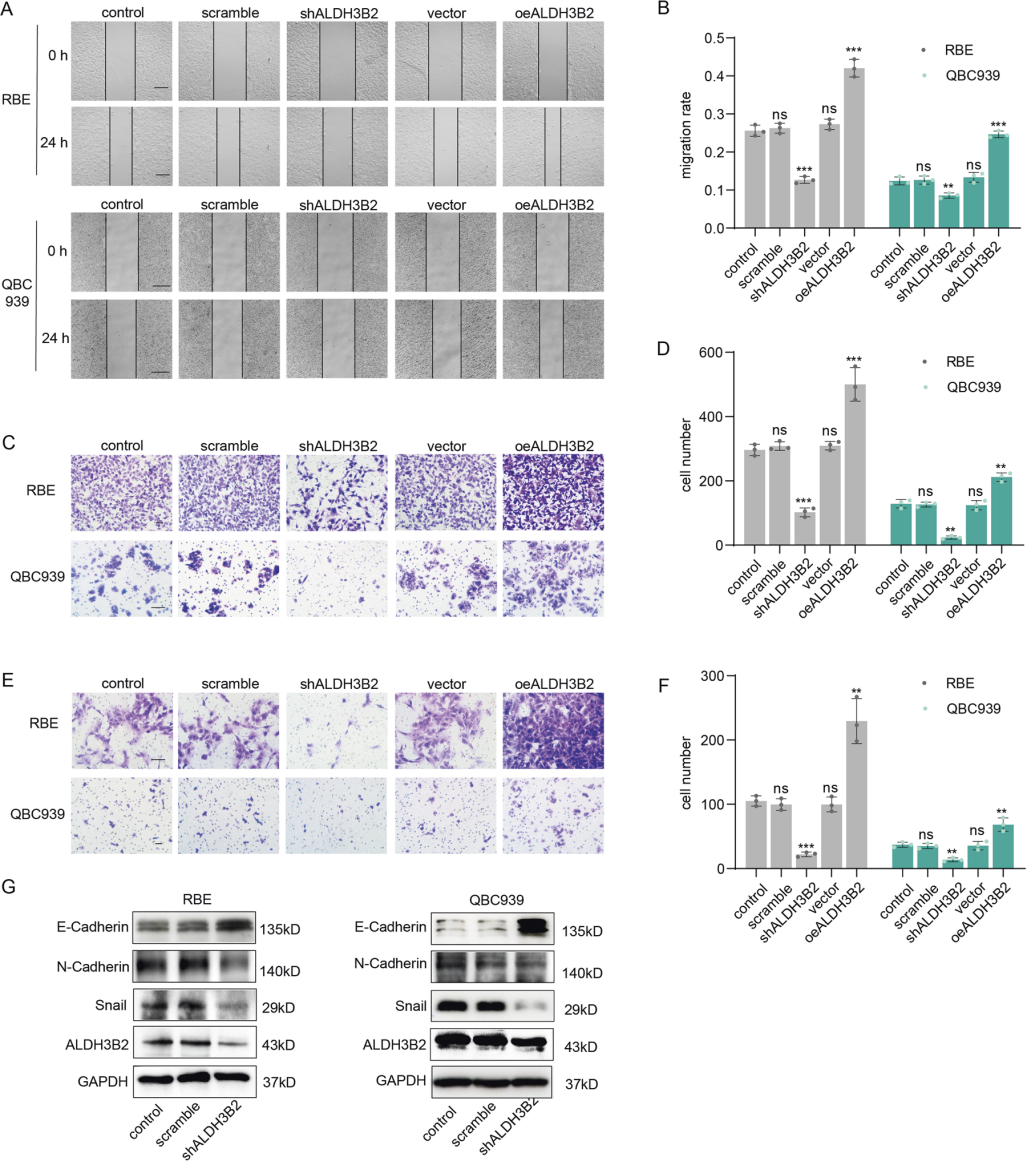
ALDH3B2 regulates cell migration, invasion, and EMT of CCA in vitro. **A**, **B** Wound healing assays were performed to investigate the migration ability after ALDH3B2 knockdown or overexpression in RBE (upper) and QBC939 (lower) cells. Cells transfected with scrambled shRNA, shALDH3B2, or ALDH3B2 overexpression plasmids were cultured for 24 h after scratching (**A**). The percentage of wound closure for cells with ALDH3B2 knockdown or overexpression was analyzed (**B**). Knockdown of ALDH3B2 inhibited the wound healing rate, while overexpression of ALDH3B2 promoted the wound healing rate. Scale bar: 500 μm. **C**, **D** Transwell assays without matrigel were used to evaluate the migration ability of RBE and QBC939 cells after ALDH3B2 knockdown or overexpression. **C** Representative images of migrated cells of RBE (upper) and QBC939 (lower) cells. **D** The statistical analysis of migrated cells with no shRNA (control), scrambled shRNA, shALDH3B2, and ALDH3B2 overexpression plasmid of RBE (left) and QBC939 (right) cells. Knockdown of ALDH3B2 inhibited the migration ability of RBE and QBC939 cells, while overexpression of ALDH3B2 promoted the migration ability of RBE and QBC939 cells. Scale bar: 100 μm. **E**, **F** Transwell assays with matrigel were used to evaluate the migration ability of RBE and QBC939 cells after ALDH3B2 knockdown or overexpression. **E** Representative images of migrated cells of RBE (upper) and QBC939 (lower) cells. **F** The statistical analysis of migrated cells with no shRNA (control), scrambled shRNA, shALDH3B2, vector, and ALDH3B2 overexpression plasmid of RBE (left) and QBC939 (right) cells. Knockdown of ALDH3B2 inhibited the invasion ability of RBE and QBC939 cells, while overexpression of ALDH3B2 promoted the invasion ability of RBE and QBC939 cells. Scale bar: 100 μm. Statistical significance between subgroups was assessed with Student’s *t* test. ns means not significant; ** and *** represent *p* < 0.01 and *p* < 0.001, respectively. **G** EMT biomarkers were detected after ALDH3B2 knockdown in RBE cells (left) and QBC939 cells (right). EMT was inhibited after ALDH3B2 knockdown in RBE cells and QBC939 cells.

Wound healing and transwell assays were then performed to detect the effect of ALDH3B2 on cell migration (Fig. 3A–D). Knockdown of ALDH3B2 notably attenuated the migration ability of RBE and QBC939 cells while overexpression of ALDH3B2 significantly promoted the migration ability of RBE and QBC939 cells. Matrigel transwell assay was also performed to examine the effect of ALDH3B2 on the invasion ability of the cholangiocarcinoma cells. Knockdown of ALDH3B2 attenuated the invasion ability of RBE and QBC939 cells while overexpression of ALDH3B2 significantly promoted the invasion ability of RBE and QBC939 cells (Fig. 3E, F). EMT-associated molecule markers were also examined in ALDH3B2 knockdown RBE and QBC939 cells. Knockdown of ALDH3B2 promoted the expression of E-cadherin and inhibited the expression of N-cadherin and snail when compared to the control cells in RBE and QBC939 cell lines (Fig. 3G and Supplemental Fig. 3). All these results indicated that ALDH3B2 played an essential role in the regulation of the cell migration, invasion, and EMT of cholangiocarcinoma.

### ALDH3B2 regulates the metastasis potency of CCA in vivo

**Fig. 4:**
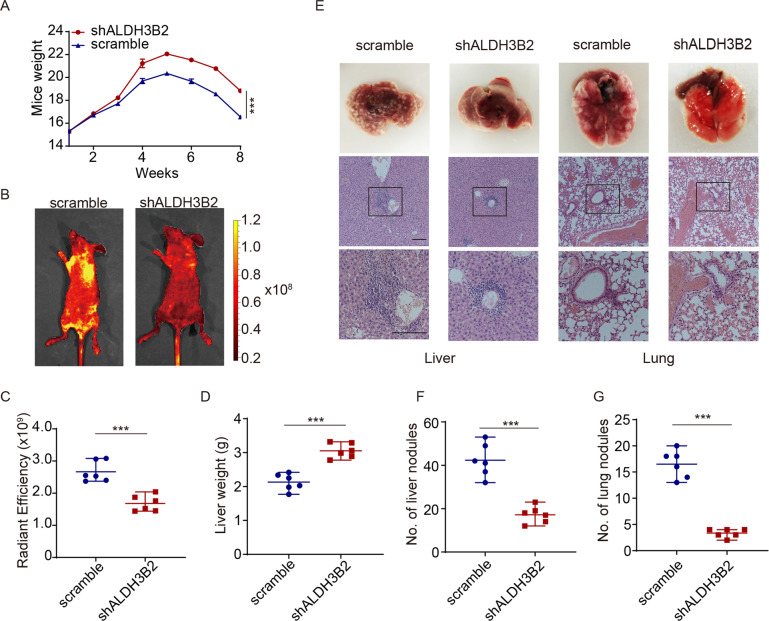
Knockdown of ALDH3B2 inhibited CCA cell metastasis ability in vivo. **A** ALDH3B2 knockdown cells and scramble cells were injected via the tail vein of BALB/c nude mice. The body weights of mice injected with the scramble and shALDH3B2 cells were measured weekly. Mice injected with ALDH3B2 knockdown cells afforded less tumor burden. **B** Metastatic models were established by tail vein injection of scramble or ALDH3B2-knockdown RBE cells. The tumor metastases were monitored by a live imaging system. **C** The statistical analysis of radiant efficiency of in vivo fluorescence in mice injected with scramble or ALDH3B2 knockdown cells (*n* = 6). **D** Statistical analysis of liver weight in mice injected with scramble and shALDH3B2 cells. **E** Representative gross specimens (upper panel) and HE staining (middle and lower panel) of metastasis lesions in liver and lung. Scale bar: 500 μm. **F**, **G** Statistical analysis of numbers of metastatic nodules in liver (**F**) and lungs (**G**) of mice. **p < 0.01, ***p < 0.001.

The function of ALDH3B2 in regulating metastasis was then examined by tail vein injection of stable ALDH3B2-knockdown or scramble RBE cells into the BALB/c nude mice. The mice injected with ALDH3B2 knockdown RBE cells had a heavier body weight, reflecting that ALDH3B2 knockdown attenuated the systemic metastasis of CCA (Fig. 4A). In vivo fluorescence imaging revealed that the ALDH3B2 knockdown group exhibited less radiant efficiency in livers and lungs (Fig. 4B, C). The liver weight of the scramble and ALDH3B2 knockdown group was also measured and the results indicated that knockdown of ALDH3B2 showed higher liver weight (Fig. 4D). Metastasis nodules were then evaluated on the surface of the lung and liver and finally confirmed with HE staining (Fig. 4E). As shown in Fig. 4E–G, ALDH3B2 silencing strikingly decreased the number of metastatic lesions, suggesting the important role of ALDH3B2 in regulating CCA metastasis.

### ALDH3B2 regulates ITGB1 expression and the activation of its downstream c-Jun, p38, and ERK

**Fig. 5:**
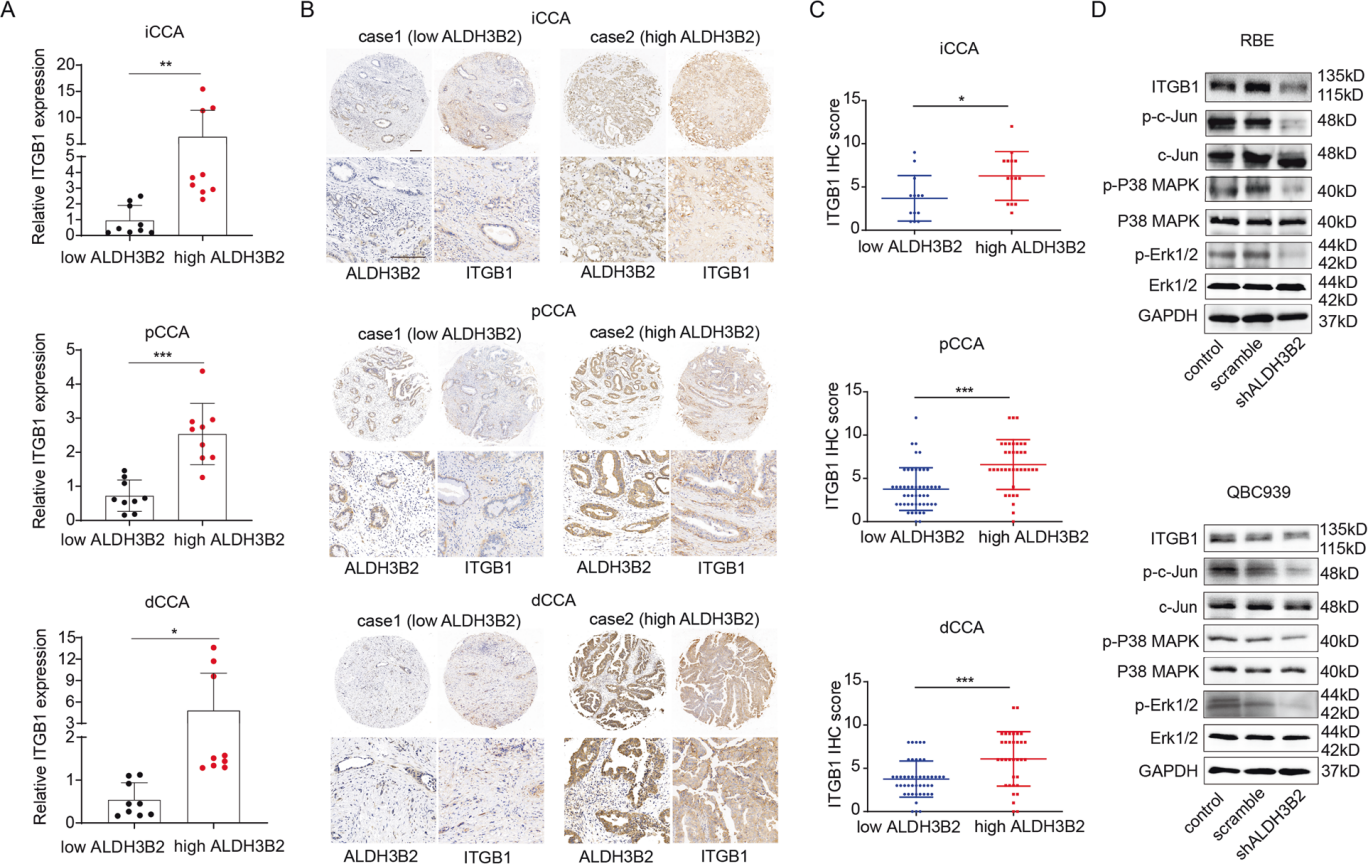
ALDH3B2 regulates the expression of ITGB1, and knockdown of ALDH3B2 inhibits ALDH3B2 downstream signaling pathway. **A** Relative mRNA expression of ITGB1 in high and low ALDH3B2 mRNA level iCCA (left), pCCA (middle) and dCCA (right) tissues (*n* = 6 for each CCA). ITGB1 was quantified by qRT-PCR. The results were analyzed using the 2^−ΔΔCT^ method with GAPDH as a reference gene. Statistical significance between groups was assessed using the Student t-test. ** represents *p* < 0.01, *** represents *p* < 0.001. **B** Typical images of immunostaining of ALDH3B2 and ITGB1 in tissue microarrays of iCCA and Case 1 (left) showed that patients with low-density staining of ALDH3B2 also presented with low-density staining of ITGB1. In contrast, patient Case 2 (right) showed that patients with low-density staining of ALDH3B2 also presented low-density staining of ITGB1. **C** Statistical analysis of the correlation between the expression of ALDH3B2 and ITGB1 in iCCA (upper), pCCA (middle), or dCCA (lower). Expression of ITGB1 and ALDH3B2 was determined by IHC score, and the data were analyzed by Student’s *t* test. Patients with high ALDH3B2 expression have relatively high expression of ITGB1. **D** Western blot analysis of the expression of ITGB1 and the phosphorylation level of its downstream signaling pathway after ALDH3B2 knockdown in RBE cells (upper) and QBC939 cells (lower).

Integrins β1(ITGB1), β2, and β3 are significant inducers of cell migration and invasion [36–42]. Integrin β1 and β3 induced EMT in breast tumors [43]. c-Jun, ERK, and p38 are the important key nodes of pathways involved in EMT mediated by ITGB1 [44–46]. We then hypothesized that ALDH3B2 promotes CCA progression by upregulating ITGB1 and activating the ITGB1 downstream signaling pathway including c-Jun, ERK1/2, and p38 MAPK pathway. The relationship between the expression of ALDH3B2 and ITGB1 at the mRNA level was first analyzed in the clinical specimen. As shown in Fig. 5A, patients with high ALDH3B2 mRNA expression levels also have high mRNA expression of ITGB1 in iCCA, pCCA, and dCCA. Correlations between ALDH3B2 protein expression and ITGB1 protein expression were then analyzed in the same TMA containing iCCA, pCCA, and dCCA. Patients with high ALDH3B2 protein expression also showed high protein expression of ITGB1 in CCA (Fig. 5B, C). Correlations between ALDH3B2 and ITGB1 was also analyzed by western blot in CCA cell lines. Expression of ITGB1 was decreased after ALDH3B2 knockdown (Fig. 5D and Supplemental Fig. 4). Moreover, the phosphorylation of c-Jun, ERK 1/2, and p38 MAPK levels was also evidently downregulated in ALDH3B2 knockdown tumor cells (Fig. 5D and Supplemental Fig. 4).

### Knockdown of ITGB1 reversed the tumor-promoting effect of ALDH3B2

**Fig. 6:**
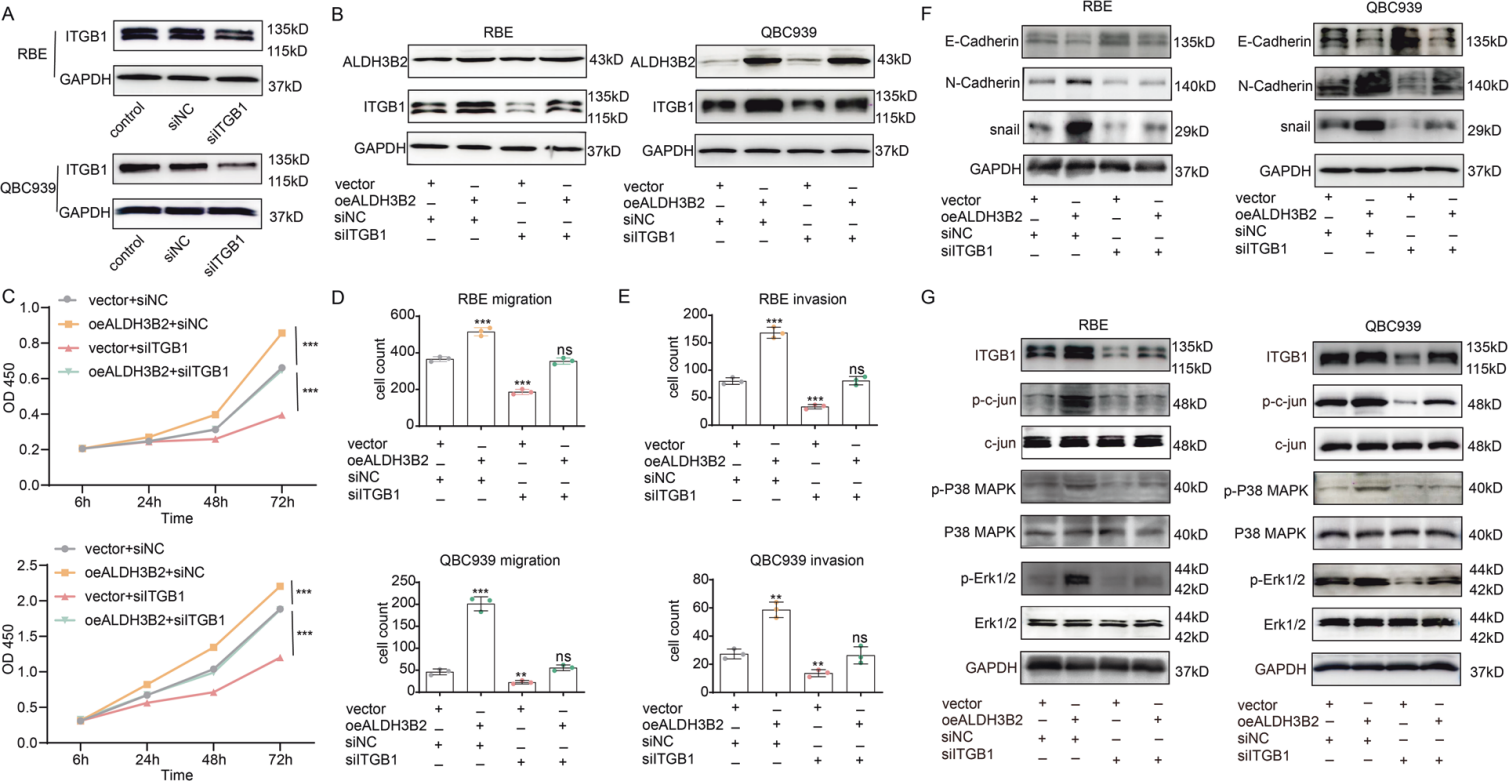
Knockdown of ITGB1 reversed the tumor-promoting effect of ALDH3B2 overexpression. **A** The knockdown efficiency of ITGB1 was determined by western blot in RBE and QBC939 cell lines. **B** Western blot analysis of the efficiency of ITGB1 knockdown in ALDH3B2-overexpressing RBE (left) and QBC939 (right) cell lines. **C** CCK-8 assays were performed to identify the alteration in cell proliferation ability after ITGB1 knockdown in ALDH3B2-overexpressing RBE (upper) and QBC939 (lower) cells. Knockdown of ITGB1 reversed the promoting effect of ALDH3B2 overexpression on the proliferation ability of RBE and QBC939 cells. **D** Transwell assays without matrigel were performed to evaluate the migration ability of RBE and QBC939 cells after ITGB1 knockdown in ALDH3B2-overexpressing RBE (upper) and QBC939 (lower) cells. Knockdown of ITGB1 reversed the promoting effect of ALDH3B2 overexpression on the migration ability of RBE and QBC939 cells. **E** Transwell assays with matrigel were performed to evaluate the invasion ability of RBE and QBC939 cells after ITGB1 knockdown in ALDH3B2-overexpressing RBE (upper) and QBC939 (lower) cells. Knockdown of ITGB1 reversed the promoting effect of ALDH3B2 overexpression on the invasion ability of RBE and QBC939 cells. **F** EMT biomarkers, including E-cadherin, N-cadherin, and snail, were detected by western blot in ALDH3B2-overexpressing RBE cells (left) and QBC939 cells (right) with or without ITGB1 knockdown. EMT-promoting effect of ALDH3B2 overexpression in RBE cells and QBC939 cells was reversed by knocking down the expression of ITGB1. **G** Western blot analysis of the expression of ITGB1 and the phosphorylation level of its downstream signaling pathway in ALDH3B2-overexpressing RBE cells (left) and QBC939 cells (right) with or without ITGB1 knockdown. Knocking down the expression of ITGB1 partially reversed the effect of ALDH3B2 overexpression.

As ALDH3B2 impacts the expression of ITGB1, we further determined whether ALDH3B2 promotes CCA progression through ITGB1. The hypothesis was then testified by knocking down the expression of ITGB1 in ALDH3B2 overexpression cells. The knockdown efficiency of ITGB1 was determined by western blot (Fig. 6A). Knockdown of ITGB1 has also been established in ALDH3B2 control and overexpression RBE and QBC939 cell lines (Fig. 6B). As shown in Fig. 6C, overexpression of ALDH3B2 promoted the proliferation ability of RBE and QBC939 cell lines, while knockdown of ITGB1 inhibited their proliferation ability. Meanwhile knockdown of ITGB1 eliminated the promoting effect of ALDH3B2 on cell proliferation. Similar effects have been observed in the transwell migration and invasion assay. Knockdown of ITGB1 inhibited the migration and invasion-promoting effect induced by ALDH3B2 overexpression (Fig. 6D, E and Supplemental Fig. 5). The effect of ITGB1 knockdown on EMT-associated molecule markers was also examined in ALDH3B2-overexpressing RBE and QBC939 cells. Overexpression of ALDH3B2 inhibited the expression of E-cadherin and promoted the expression of N-cadherin and snail when compared to the control cells in RBE and QBC939 cell lines while knockdown of ITGB1 in ALDH3B2-overexpressing cells reversed the effect of ALDH3B2 on the expression of E-cadherin, N-cadherin, and snail (Fig. 6F). The effect of ITGB1 knockdown on the phosphorylation level of c-Jun, p38 MAPK, and ERK was also analyzed in ALDH3B2-overexpressing RBE and QBC939 cells. As shown in Fig. 6G, knocking down of ITGB1 downregulated the phosphorylation level of c-Jun, p38 MAPK, and ERK and partially reversed the effect of ALDH3B2 overexpression. These results indicate that ALDH3B2 promotes CCA progression by promoting the expression of ITGB1.

### Double expression of ALDH3B2 and ITGB1 is a strong biomarker of CCA prognosis

**Fig. 7:**
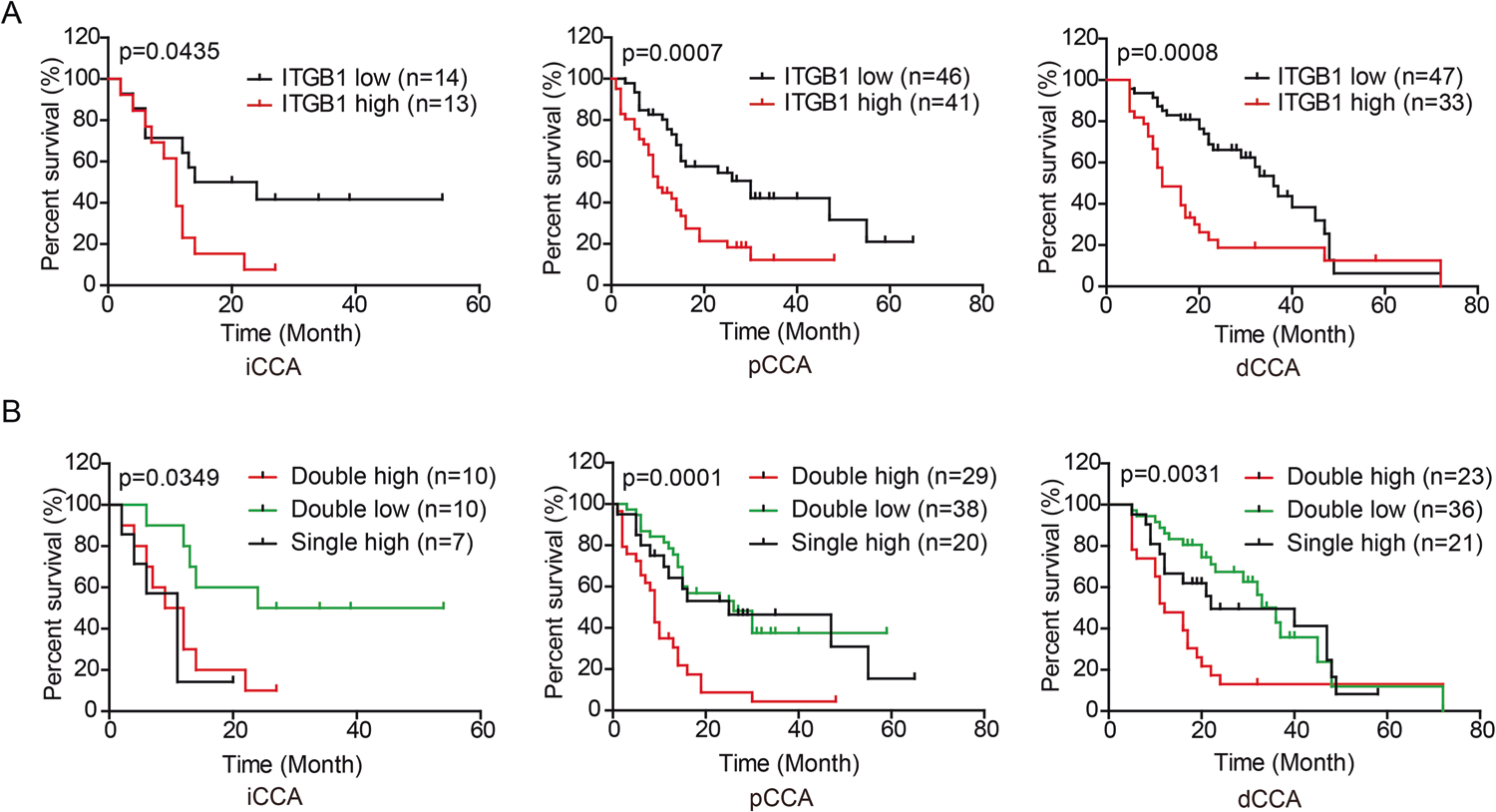
Prognostic role of ITGB1 and double expression of ALDH3B2 and ITGB1 in predicting CCA prognosis. **A** The overall survival curves of patients with iCCA (left), pCCA (middle), and dCCA (right) were stratified by ITGB1 expression. High ITGB1 expression indicates poor patient overall survival. **B** Overall survival curves of patients with iCCA (left), pCCA (middle), and dCCA (right) were further analyzed by stratifying the patients into subgroups with the double high expression, single high expression, and dual low expression of ALDH3B2 and ITGB1. A log-rank test calculated data. Double high expression of ALDH3B2 and ITGB1 was more potent in predicting patient prognosis.

As ITGB1 was identified as a downstream effector, we also analyzed whether ITGB1 expression has a prognostic role in our patient cohort. As shown in Fig. 7A, high ITGB1 expression correlates with poor patient prognosis in iCCA (*p* = 0.0435), pCCA (*p* = 0.0007) and dCCA (*p* = 0.0008). The duplicated effect of double-positive expression of ALDH3B2 and ITGB1 in predicting patient prognosis was also analyzed. As shown in Fig. 7B, patients with double-positive expression of ALDH3B2 and ITGB1 exhibits even poorer prognosis in iCCA, pCCA, and dCCA. These results indicate that double-positive expression of ALDH3B2 and ITGB1 is a strong biomarker of CCA prognosis.

## Discussion

ALDHs are enzymes that participate in important cellular activities including aldehyde detoxification [26]. Direct or indirect ALDH inhibition, using specific inhibitors, has shown a reduction in ALDH activity, resulting in the loss of stem cell traits, reduction of cell proliferation, invasion, and drug sensitization [47]. However, whether ALDHs participate in the oncogenesis and progression of cholangiocarcinoma has not yet been evaluated. In this study, we compared the expression of 19 ALDHs in hilar cholangiocarcinoma and paired peritumor tissues by mRNA microarray. The results showed that ALDH3B1 and ALDH3B2 were highly expressed in hilar cholangiocarcinoma, which indicated the important role of ALDH3 family in the development of cholangiocarcinoma. Further survival analysis showed that high ALDH3B2 indicated poor patient prognosis, while ALDH3B1 had no prognostic value. In perihilar cholangiocarcinoma, higher ALDH3B2 expression was associated with higher T stage, higher M stage, higher incidences of neural invasion and higher serum CA19-9 while high ALDH3B2 expression was associated with high serum CEA in iCCA or dCCA. Moreover, high expression of ALDH3B2 was an independent prognostic factor for iCCA and pCCA. To our knowledge, this is the first time to evaluate the prognostic value of ALDH3B2 in cholangiocarcinoma. These results also reveal that ALDH3B2 is an important oncogene, which is worthy of further study.

ALDH3B2 belongs to the ALDH3 subfamily of the ALDH family [48]. Mammalian ALDH3 genes (ALDH3A1, ALDH3A2, ALDH3B1, and ALDH3B2) encode enzymes of peroxidic and fatty aldehyde metabolism [49]. The gene polymorphism of ALDH3B2 is associated with the susceptibility of colorectal cancer and esophageal squamous cell carcinoma [50, 51]. ALDH3B2 participates in an eleven metabolic gene signature-based prognostic model for clear cell renal cell carcinoma and a gene expression signature-based nomogram model in the prediction of breast cancer bone metastases [52, 53]. We first studied the effect of ALDH3B2 knockdown or overexpression on the proliferation ability of cholangiocarcinoma cells. Similar to previous studies in the ALDH family, we observed that inhibition of ALDH3B2 expression can inhibit the proliferation and clonogenic ability of cholangiocarcinoma cells by inducing G1 phase arrest [54–56].

Nerve invasion, metastasis along the bile duct, and lymph node metastasis are important characteristics of cholangiocarcinoma and factors of poor prognosis. In our study, we analyzed the effect of ALDH3B2 expression on the metastasis ability of cholangiocarcinoma cells. The results showed that inhibition of ALDH3B2 expression inhibited the migration and invasion ability of cholangiocarcinoma cells. The role of ALDH3B2 on the EMT of cholangiocarcinoma cells was also evaluated. The results revealed that knockdown of ALDH3B2 promoted the expression of E-cadherin and inhibited the expression of N-cadherin and snail, indicating that ALDH3B2 may also promote the progression of cholangiocarcinoma by promoting EMT.

Tail vein injection is an important model to detect the metastatic ability of cholangiocarcinoma cells [5]. We also studied the effect of ALDH3B2 knockdown on the metastatic ability of cholangiocarcinoma cells by tail vein injection. The results showed that ALDH3B2 knockdown inhibited the liver metastasis and lung metastasis of cholangiocarcinoma cells, and reduced the tumor burden of mice. These results, for the first time, elucidated the important regulatory role of ALDH3B2 in cell metastasis at the cellular level and indicate that the targeted inhibition of ALDH3B2 function may inhibit the metastasis of cholangiocarcinoma cells and prolong the survival of patients.

Our previous study revealed that ITGB1 was an important metastasis regulating gene [42]. High integrin expression can induce invasion and EMT. Integrin β1 enhanced EMT by activating the FAK-Akt signaling pathway in gefitinib-resistant non-small cell lung cancer tumor cells [57]. Integrin αvβ3 was upregulated and contributed to the migration of lung cancer cells by activating the FAK-Akt and NF-kappaB pathway in A549 lung cancer cells [58]. Cordycepin significantly suppressed the expression of integrin α3, integrin α6, and integrin β1, which are crucial interacting partners of FAK in contributing to EMT [59]. Therefore, we examined whether ALDH3B2 had an impact on the expression of ITGB1 and the phosphorylation level of the ITGB1 downstream signaling pathway including c-Jun, ERK 1/2, and p38 MAPK. We found that the expression of ITGB1 correlates with the expression of ALDH3B2 at mRNA and protein level in iCCA, pCCA, and dCCA specimens. Knockdown of ALDH3B2 downregulated the expression of ITGB1 and the phosphorylation level of ITGB1 downstream signaling pathway. Meanwhile, knockdown of ITGB1 in ALDH3B2-overexpressing CCA cell lines reversed the promoting effect of ALDH3B2 overexpression on cell proliferation, migration, invasion, and EMT. Knockdown of ITGB1 also partially reversed the promoting effect of ALDH3B2 overexpression on the phosphorylation level of c-Jun, ERK 1/2, and p38 MAPK. These results indicate that ALDH3B2 promotes the progression of cholangiocarcinoma by upregulating the expression of ITGB1 and promoting the phosphorylation level of the ITGB1 downstream signaling pathway. These results for the first time established a regulating role of ALDH3B2 on the expression of ITGB1 and elucidated the potential molecular mechanism of ALDH3B2 on the progression of malignant tumors.

There are also some points need further elucidation. First, ALDH3B2 belongs to the ALDH family. Most ALDH family members function by dehydrogenating aldehyde. ALDH1 was initially identified to be a drug-resistant gene by dehydrogenating cyclophosphamide intermediate [60]. ALDH2 is essential participant in the redox reaction of ethanol and the endogenous aldehyde product released by lipid peroxidation [61]. ALDH1 and ALDH2 are also biomarkers of cancer stem cells (CSCs) and related to cancer cells’ proliferation, metastasis, and multidrug resistance (MDR) [24, 61] while the detailed mechanism remains to be elucidated. Our results indicate that ALDH3B2 has considerable research value. However, as an enzyme, whether the metabolites of ALDH3B2 are involved in influencing downstream gene expression deserves further study. Secondly, although our results revealed that both ALDH3B2 and ITGB1 are prognostic factors of iCCA, the sample size of iCCA is relatively small (n = 27) and the conclusion should be further validated by a larger patient cohort.

In conclusion, our findings demonstrated that high expression of ALDH3B2 on cholangiocarcinoma promotes tumor metastasis by upregulating integrin β1 and upregulating the phosphorylation level of c-Jun, p38, and ERK. ALDH3B2 together with ITGB1 are prognostic factors of iCCA, pCCA, and dCCA. More importantly, double-positive expression of ALDH3B2 and ITGB1 is a strong biomarker of CCA prognosis. These results indicate that ALDH3B2 can be regarded as a potential poor prognostic marker for cholangiocarcinoma and that ALDH3B2 may serve as a promising treatment target.

## Supplementary information

Reproducibility checklist

Supplemental material

## Data Availability

The datasets generated and/or analyzed during the current study are available from the corresponding author on reasonable request.
