# Supplementary material for: Aldehyde dehydrogenase 3B2 promotes the proliferation and invasion of cholangiocarcinoma by increasing Integrin Beta 1 expression
Source: Cell Death Dis. 2021 Dec 14;12(12):1158. doi: 10.1038/s41419-021-04451-8 (PMC8671409; doi:10.1038/s41419-021-04451-8)
Supplement: Supplementary file 2 — Supplemental material [file 41419_2021_4451_MOESM2_ESM.docx]

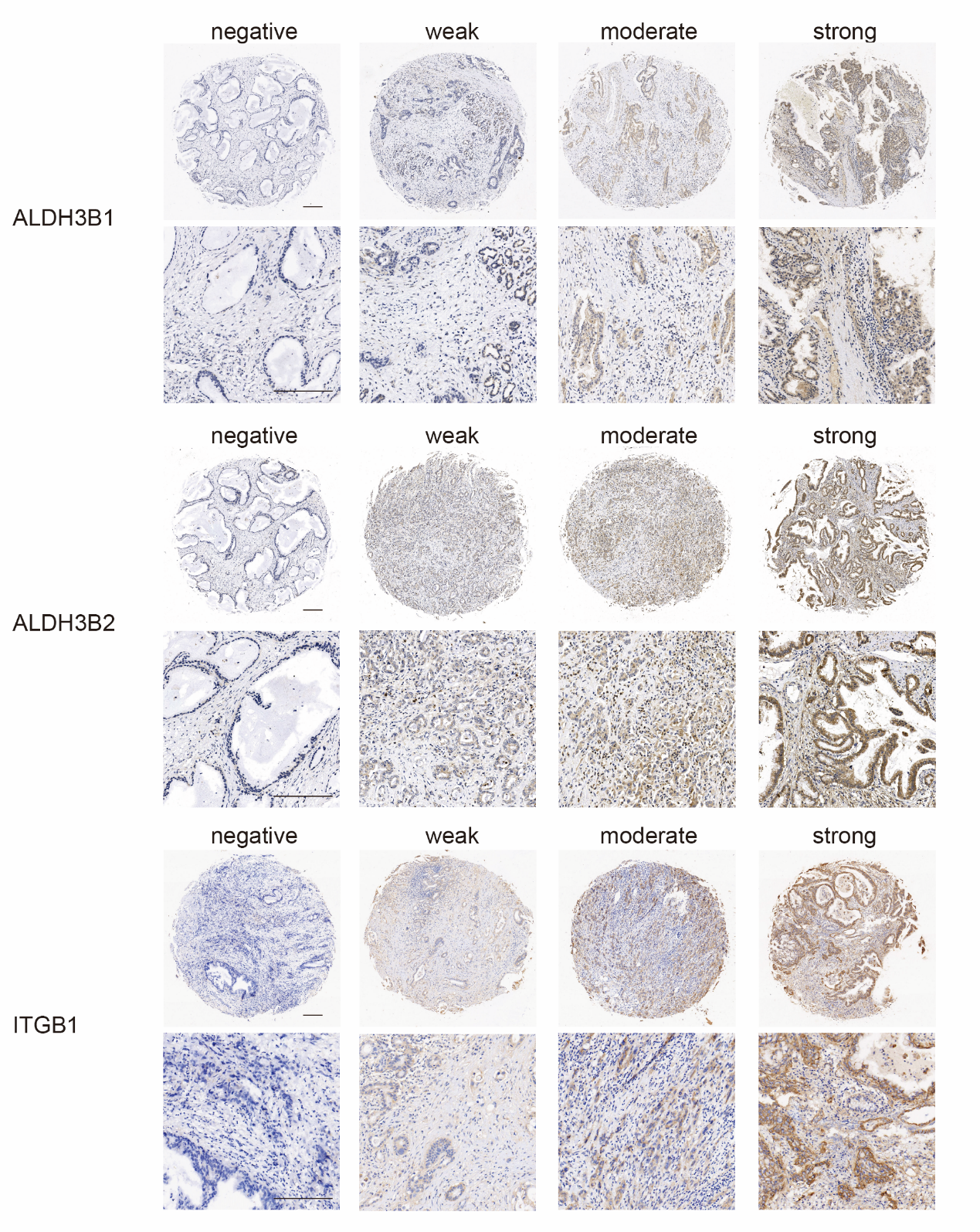


Supplemental Figure 1: Typical images indicating negative, weak, moderate and strong positive expression of ALDH3B1, ALDH3B2 and ITGB1 which is applied for IHC scoring.


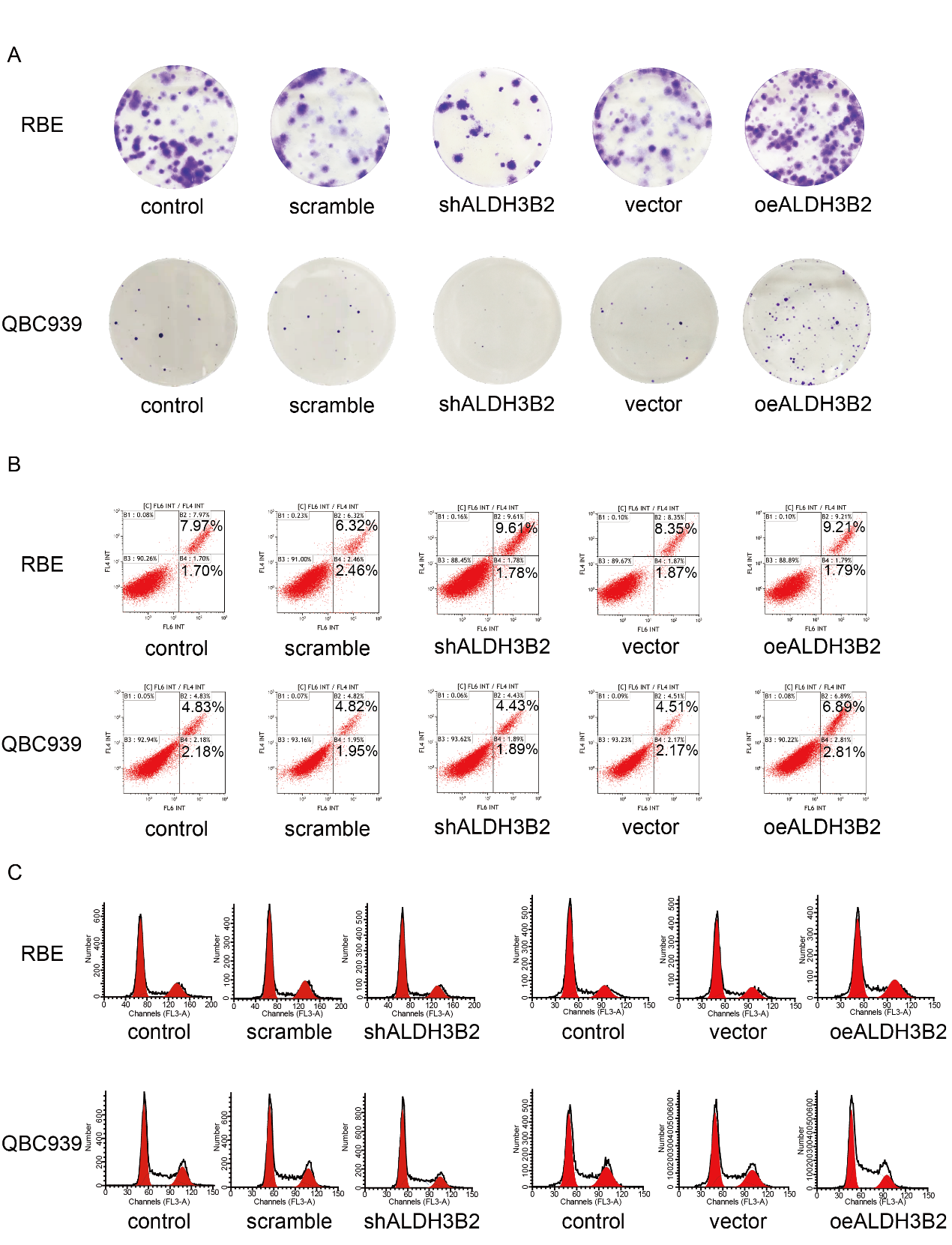


Supplemental Figure 2: ALDH3B2 promotes the cell proliferation ability of CCA by promoting the G1/S phase transition *in vitro*.

1. Typical images of clone formation of cells with ALDH3B2 knockdown or overexpression compared with their vector controls in RBE (upper) and QBC939 cells (lower).
2. Typical images of flow cytometry performed for detecting apoptotic rates in ALDH3B2-overexpressing and knockdown RBE (upper) and QBC939 cells (lower) cells. ALDH3B2 did not affect the apoptotic rates in these two cell lines. ns: no significance.
3. Typical images of cell cycle detected with flow cytometry after ALDH3B2 knockdown (left) or overexpression (right) in RBE (upper) and QBC939 cells (lower).


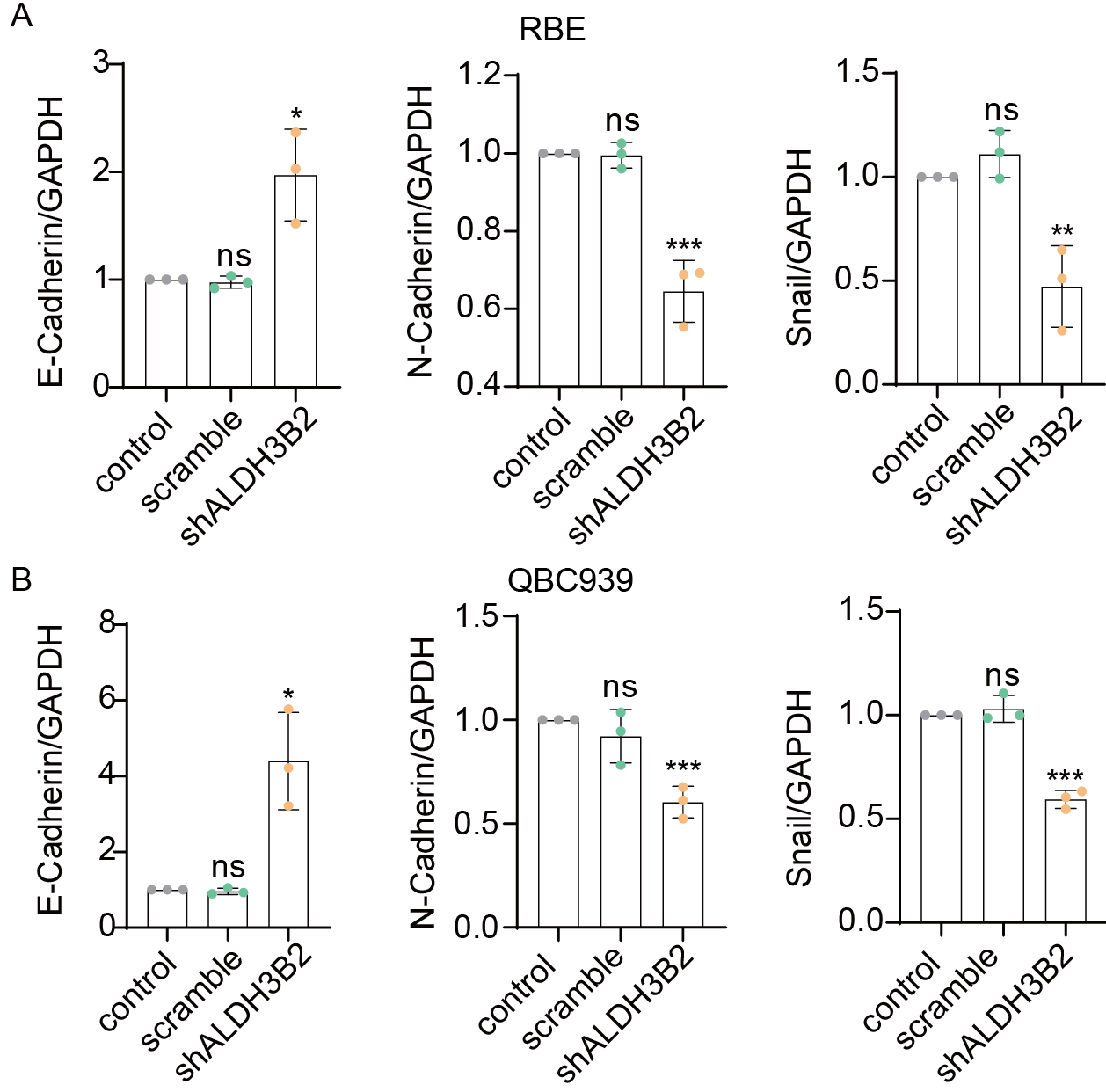


Supplemental Figure 3: Statistical analysis of the expression of ITGB1 and the phosphorylation level of c-Jun, ERK1/2 and p38 MAPK in RBE cells (A) and QBC939 cells (B).


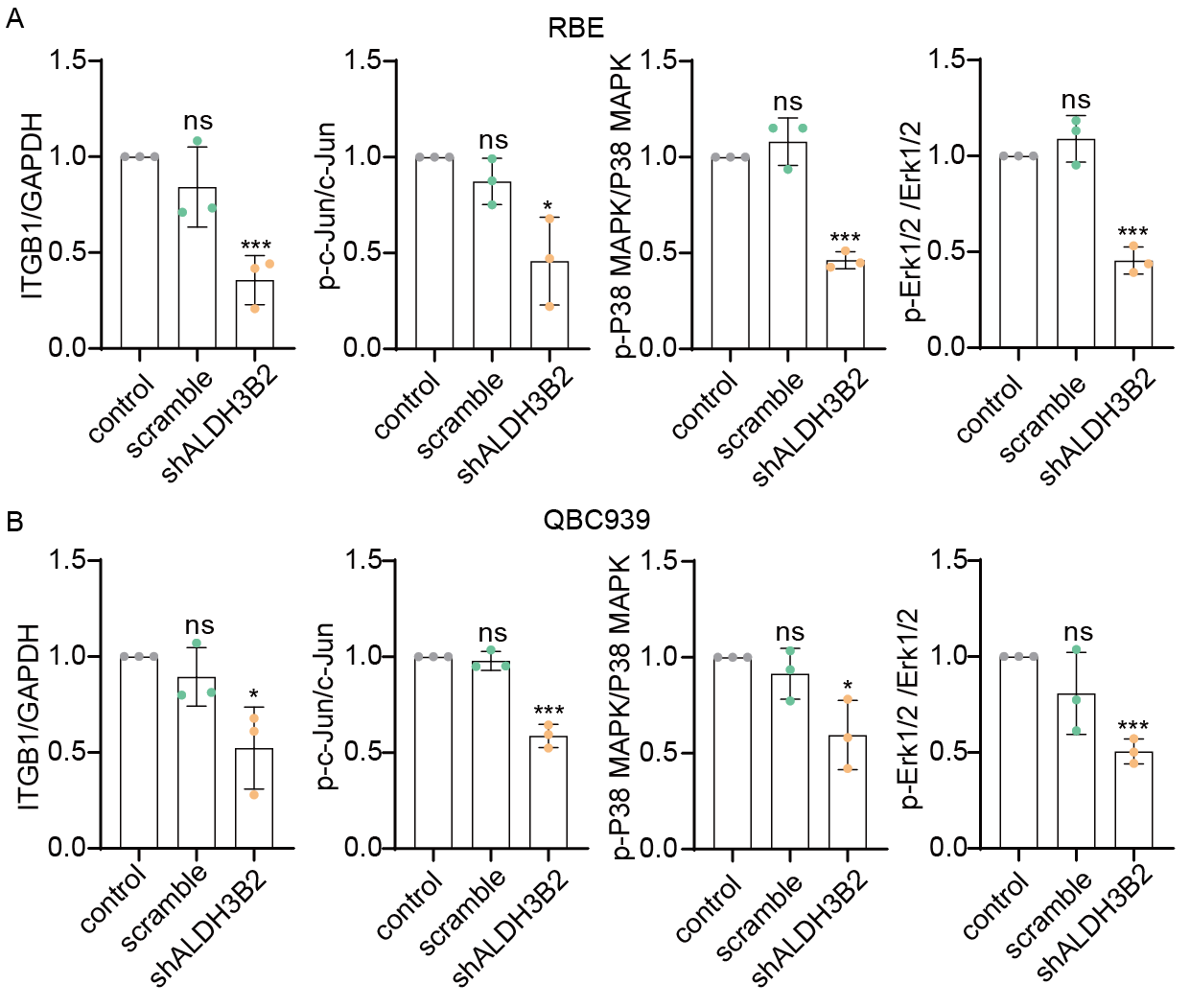


Supplemental Figure 4: Statistical analysis of the expression of EMT biomarkers including E-cadherin, N-cadherin and snail after ALDH3B2 knockdown in RBE cells (A) and QBC939 cells (B).


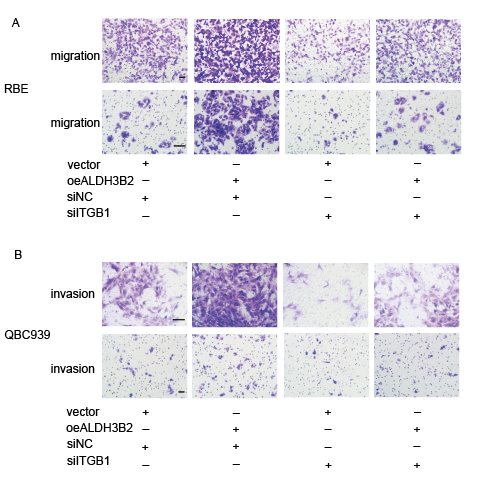


Supplemental Figure 5: Typical images of cell migration and invasion ability assay. (A) Transwell assays without matrigel were performed to evaluate the migration ability of RBE and QBC939 cells after ITGB1 knockdown in ALDH3B2 overexpressing RBE (upper) and QBC939 (lower) cells.

(B) Transwell assays with matrigel were performed to evaluate the invasion ability of RBE and QBC939 cells after ITGB1 knockdown in ALDH3B2 overexpressing RBE (upper) and QBC939 (lower) cells.

Supplemental Table 1 The manufacturer and catalog number information of the applied antibodies in this study.

| Antibodies | Source | Identifier |
| --- | --- | --- |
| ALDH3B2 | proteintech | Cat. No.15746-1-AP |
| ALDH3B1 | proteintech | Cat. No. 19446-1-AP |
| Integrin β1 | Cell Signaling Technology (CST) | Cat. No. 9699T |
| phospho-Erk1/2 | CST | Cat. No. 4370S |
| Erk1/2 | CST | Cat. No. 4695S |
| c-Jun | CST | Cat. No. 9165T |
| phospho-c-Jun | CST | Cat. No. 3270T |
| p38 MAPK | CST | Cat. No. 8690T |
| Phospho-p38 MAPK | CST | Cat. No. 4511T |
| N-Cadherin | CST | Cat. No. 13116S |
| E-Cadherin | CST | Cat. No. 3195S |
| Snail | CST | Cat. No. 3879S |
| GAPDH | Santa Cruz | Cat. No. sc-47724 |
